# Correction: Silencing GOLGA8B inhibits cell invasion and metastasis by suppressing STAT3 signaling pathway in lung squamous cell carcinoma

**DOI:** 10.1042/CS-2022-0128_COR

**Published:** 2024-08-09

**Keywords:** GOLGA8B, lung squamous cell carcinoma, metastasis, STAT3

The authors of the original article “Silencing GOLGA8B inhibits cell invasion and metastasis by suppressing STAT3 signaling pathway in lung squamous cell carcinoma” (DOI: 10.1042/CS20220128) would like to correct the Funding statement in their paper. The authors state there is an error in the funding number for “Hunan Provincial Natural Science Foundation of China (2022JJ40942)”. The correct funding number should read: “Hunan Provincial Natural Science Foundation of China (2022JJ30942)”. The requested correction has been assessed and agreed by the Editorial Board.

